# Draft Genome Sequence of Marine Actinomycete *Streptomyces* sp. Strain NTK 937, Producer of the Benzoxazole Antibiotic Caboxamycin

**DOI:** 10.1128/genomeA.00534-14

**Published:** 2014-07-03

**Authors:** Carlos Olano, Carolina Cano-Prieto, Armando A. Losada, Alan T. Bull, Michael Goodfellow, Hans-Peter Fiedler, Carmen Méndez, José A. Salas

**Affiliations:** a Departamento de Biología Funcional e Instituto Universitario de Oncología del Principado de Asturias (I.U.O.P.A.), Universidad de Oviedo, Oviedo, Spain; b School of Biosciences, University of Kent, Canterbury, Kent, United Kingdom; c School of Biology, University of Newcastle, Newcastle upon Tyne, United Kingdom; d Mikrobiologisches Institut, Universität Tübingen, Tübingen, Germany

## Abstract

*Streptomyces* sp. strain NTK 937 is the producer of the benzoxazole antibiotic caboxamycin, which has been shown to exert inhibitory activity against Gram-positive bacteria, cytotoxic activity against several human tumor cell lines, and inhibition of the enzyme phosphodiesterase. In this genome announcement, we present a draft genome sequence of *Streptomyces* sp. NTK 937 in which we identified at least 35 putative secondary metabolite biosynthetic gene clusters.

## GENOME ANNOUNCEMENT

*Streptomyces* sp. strain NTK 937 was isolated from an Atlantic Ocean deep-sea sediment core collected in 2001 at the southern edge of the Saharan debris flow near the Canary Islands, at a depth of 3,814 m (1). This strain produces the benzoxazole antibiotic caboxamycin (1), whose core structure is part of other cytotoxic benzoxazoles, such as UK-1 (2), AJI9561 (3), and nataxazole (4). The cytotoxic activity of caboxamycin is lower than that of UK-1, but in contrast to UK-1 (2), it shows antibiotic activity against Gram-positive bacteria (1). In addition, caboxamycin was found to be an inhibitor of phosphodiesterases, regulators of cyclic nucleotide signaling, and potential targets for treating asthmatic inflammation and chronic obstructive pulmonary disease (1).

Here, we present the draft genome sequence of the caboxamycin producer strain generated by real-time DNA sequencing using the PacBio RS II platform (5). After the sequencing runs, a total of 77,344 paired-end sequences were obtained, with a mean of 5,551 nucleotides, which gave us a total of 429.34 Mb. A *de novo* assembly was performed using a preassembly by HGAP version 2.0.2 (6) and a final assembly using the whole-genome shotgun (WGS)-Celera Assembler version 7.0 (7), followed by an assembly polishing with the Quiver algorithm (8). The final assembly resulted in 3 contigs, the largest being 7,003,153 nucleotides and the shortest 18,266 nucleotides. The initial draft genome contains 7,444,861 nucleotides at 57.67-fold coverage, with a G+C content of 71.90%. The annotation was performed using the PGAAP pipeline (9), resulting in the identification of 6,134 coding sequences, 18 rRNA operons, and 81 tRNA loci.

We searched the genome sequence of the *Streptomyces* sp. NTK 937 chromosome using the bioinformatic tool antiSMASH 2.0 (10) and found 35 predicted secondary metabolite biosynthetic gene clusters. Fourteen of these clusters contain modular enzyme-coding genes, such as genes for polyketide synthases (PKS) (4 type I and 1 type II PKS), nonribosomal peptide synthetases (NRPS) (5 clusters), or hybrid PKS-NRPS (4 clusters). Thirteen clusters might be involved in the biosynthesis of other peptides or amino acid compounds, such as siderophores (1 cluster), ribosome-derived peptides (3 lantipeptides and 8 bacteriocins), and ectoine (1 cluster). The remaining eight gene clusters might be involved in the biosynthesis of terpenes (6 clusters), butyrolactone (1 cluster), and melanin (1 cluster).

The *Streptomyces* sp. NTK 937 genome sequence will allow the identification of a caboxamycin biosynthesis gene cluster for investigation of its pathway and generation of novel derivatives using combinatorial biosynthesis and genetic tools gathered from other benzoxazole pathways. Furthermore, this chromosome is a good candidate for genome mining of new gene clusters and other bioactive compounds.

### Nucleotide sequence accession numbers.

This whole-genome shotgun project has been deposited in DDBJ/EMBL/GenBank under the accession no. JJOB00000000. The version described in this paper is the first version, JJOB01000000.
